# A single nucleotide polymorphism in the 3′-UTR of *STAT3* regulates its expression and reduces risk of pancreatic cancer in a Chinese population

**DOI:** 10.18632/oncotarget.11607

**Published:** 2016-08-25

**Authors:** Beibei Zhu, Ying Zhu, Jiao Lou, Juntao Ke, Yi Zhang, Jiaoyuan Li, Yajie Gong, Yang Yang, Jianbo Tian, Xiating Peng, Danyi Zou, Rong Zhong, Jing Gong, Jiang Chang, Lu Li, Xiaoping Miao

**Affiliations:** ^1^ State Key Laboratory of Environment Health (Incubation), MOE (Ministry of Education) Key Laboratory of Environment and Health, Ministry of Environmental Protection Key Laboratory of Environment and Health (Wuhan), and Department of Epidemiology and Biostatistics, School of Public Health, Tongji Medical College, Huazhong University of Science and Technology, Wuhan, China; ^2^ Second Affiliated Hospital, Shantou University Medical College, Shantou, Guangdong, China

**Keywords:** pancreatic cancer, STAT3, genetic variants, susceptibility, case-control study

## Abstract

Pancreatic cancer (PC) is one of the deadliest solid malignancies carrying a gloomy 5-year survival rate less than 5%. The signal transducer and activator of transcription 3 (*STAT3*) is a common transcriptional regulator, whose aberrant expression has been widely found in human cancers, including PC. Our current study aimed to illustrate the roles of common variants, in the three prime untranslated region (3′UTR) of *STAT3*, in modifying the risk of PC through two-stage case-control studies integrating biological experiments. We first explored the associations between two common variants (rs1053004 and rs1053005) and PC risk in 774 PC cases and 777 controls. Only rs1053004 T > C showed a significant association with a reduced risk of PC with an odds ratio (OR) and 95% confidence interval (CI) of 0.85 (0.74–0.98). Then we attempted to validate the association in another 940 cases and 1398 controls, and the significant association persisted with OR (95%CI) of 0.86 (0.76–0.97). Dual luciferase reporter gene assays indicated that C allele conferred a higher expression of *STAT3* in three PC cell lines including Panc-1 (*P* = 3.0 × 10^−3^), BxPC-3 (*P* = 6.7 × 10^−5^) and SW1990 (*P* = 4.0 × 10^−3^). In conclusion, the current study provided evidence that rs1053004 T > C in 3′UTR of *STAT3* may decrease the risk of PC through up-regulating the gene expression.

## INTRODUCTION

Pancreatic cancer (PC) is the fifteenth most common cancer in the world with extremely low 5-year survival rate at only ~5% [1]. Despite decades of efforts, there are still few early detection methods and effective treatments. These grim facts highlight pressing need to identify the risk factors of PC for primary prevention and targeted therapy. Many environmental factors might influence an individual's PC risk [2, 3], among which cigarette smoking has been confirmed as the surest to date. Both current and former smokers have higher risk than non-smokers [4]. Approximately 25% of PC cases are attributable to smoking [5, 6]. Individuals with family history are prone to PC and risk rises with the increasing number of affected first-degree relatives [7], which indicates inherited genetic background may impact on susceptibility to PC. However, only a small part of sporadic PCs can be explained by the genetic variants identified by recent genome wide association studies [8–14], while some true susceptibility loci with moderate *P* values might have been overlooked due to stringent *P*-value threshold [15]. Therefore, the genetic susceptibility to sporadic PC is definitely worth exploring.

STAT3 is a member of the STAT protein family, which are mainly phosphorylated by the receptor or non-receptor associated kinases, in response to cytokines and growth factors, and then form homo- or heterodimers that translocate to the cell nucleus where they act as transcription activators. They regulate cytokine-dependent inflammation and immunity, determining whether immune responses in the tumor microenvironment promote or inhibit cancer [16].

STAT3 plays a central role in many inflammatory pathways including nuclear factor kappa-light-chain-enhancer of activated B cells (NF-κB) and interleukin-6 (IL-6)-GP130-Janus kinase (JAK) pathways [16]. Considering that deregulation of transcriptional control is a central characteristic of cancers [17], and inflammation is a defined cancer-causing factor [18], it is reasonable to presume that alteration of *STAT3* may be involved in the process of carcinogenesis. However, the roles it plays may be complicated and two-sided. In some circumstances, *STAT3* is regarded as an oncogene [19–24], while in others its tumor suppressing and prognosis ameliorating effects have also been identified [25–28].

In pancreatic ductal adenocarcinomas (PDACs), constitutive activation of STAT3 by phosphorylation of Tyr705 has been reported in 30% to 100% of human tumor specimens, as well as in many PDAC cell lines [29]. Notably, epithelial Stat3 is required particularly during the progression of PanIN to PDAC, but not in the initiation of PanIN in mouse model [30]. Based on these findings, we hypothesized that common variants in STAT3 may contribute to the susceptibility to PC. Since 3′UTR often contains regulatory elements that post-transcriptionally regulate gene expression, we aimed at finding functional variants in this region. Thus we conducted two stage case-control studies to evaluate the correlation between two common variants (rs1053004, rs1053005) in 3′UTR of *STAT3*, which were predicted most likely to have potential function, and risk of PC. Furthermore, luciferase reporter gene assays were performed for function verification.

## RESULTS

### Characteristics of population

A total of 774 cases and 777 controls passed quality control of stage one, and 940 cases and 1398 controls were included in stage two. The characteristics of the subjects are summarized in Table 1. The majority of cases were males, with 57.4% in stage one and 79.4% in stage two. The median ages of case/control groups in two stages were 60/59 and 61/60, respectively. Cases and controls were adequately matched in sex and age in both stages.

**Table 1:** Characteristics of subjects in this study

	Stage one				Stage two			
Variables	Case *N*(%)	Control *N* (%)	χ^2^	*P*	Case *N*(%)	Control *N* (%)	χ^2^	*P*
Total	774	777			940	1398		
Sex			0.045	0.833			0.657	0.674
Male	444 (57.4)	450 (57.9)			746 (79.4)	1120 (80.1)		
Female	330 (42.6)	327 (42.1)			194 (20.6)	278 (19.9)		
Age(Median)	60	59	1.240	0.743	61	60	0.470	0.925
< 45	94 (12.1)	94 (12.1)			108 (11.5)	148 (10.6)		
45~55	163 (21.1)	159 (20.5)			184 (19.6)	277 (19.8)		
55~65	252 (32.6)	273 (35.1)			311 (33.1)	467 (33.4)		
> 65	265 (34.2)	251 (32.3)			337 (35.9)	506 (36.2)		

### Association analysis

Both of the two SNPs (rs1053004 and rs1053005) were successfully genotyped. The call rates in both stages were > 95%, and the genotypes in controls conformed to HWE (*P* > 0.05). In stage one, only rs1053004 T > C polymorphism was found to be significantly associated with a decreased PC risk (CC versus TT: odds ratio (OR) (95% confidence interval (CI)) = 0.71 (0.52–0.96); *P* = 0.025). While no significant association was found between genotypes of rs1053005 and PC risk (CC versus TT: OR (95% CI) = 0.80 (0.57–1.11); *P* = 0.176). Rs1053004 was further replicated in stage two (CC versus TT: OR (95% CI) = 0.70 (0.53–0.93); *P* = 0.013). Consistently, individuals carrying rs1053004 CC genotype had lower risk of PC compared with the TT genotype (OR (95% CI) = 0.72 (0.59–0.88); *P* = 0.002) in the combined samples. And rs1053004 also showed to be significantly associated with PC risk in additive model in both two stages (OR (95% CI) _stage one_ = 0.85 (0.73–0.98); OR (95% CI) _stage two_ = 0.86 (0.76–0.97)) and combined samples (OR (95% CI) _combined_ = 0.86 (0.78–0.94)). Two other models (dominant, recessive models) were also demonstrated in combined samples. The genotype frequencies and detailed statistical results of rs1053004 and rs1053005 are summarized in Table 2 and Supplementary Table S1, respectively. Also, results of stratified analysis of association between rs1053004 and risk of PC by gender was presented in Supplementary Table S2.

**Table 2:** Association between rs1053004 and risk of PC

Genotype	Stage one			*P*	Stage two			*P*	Combined			*P*
	Case	Control	OR^a^		Case	Control	OR^a^		Case	Control	OR^a^	
	*N* (%)	*N* (%)	(95% CI^b^)^c^		*N* (%)	*N* (%)	(95% CI^b^)^c^				(95% CI^b^)^c^	
rs1053004												
TT	345 (44.6)	310 (39.9)	1.00		414 (44.0)	564 (40.3)	1.00		759 (44.3)	874 (40.2)	1.00	
CT	330 (42.6)	341 (43.9)	0.87 (0.70–1.08)	0.202	431 (45.9)	650 (46.5)	0.90 (0.76–1.08)	0.263	761 (44.4)	991 (45.6)	0.89 (0.77–1.02)	0.085
CC	99 (12.8)	126 (16.2)	0.71 (0.52–0.96)	**0.025**	95 (10.1)	184 (13.2)	0.70 (0.53–0.93)	**0.013**	194 (11.3)	310 (14.3)	0.72 (0.59–0.88)	**0.002**
Additive			0.85 (0.73–0.98)	**0.023**			0.86 (0.76–0.97)	**0.017**			0.86 (0.78–0.94)	**0.001**
Recessive			0.76 (0.57–1.01)	0.055			0.74 (0.57–0.96)	**0.026**			0.77 (0.63–0.93)	**0.006**
Dominant			0.83 (0.68–1.01)	0.062			0.86 (0.73–1.02)	0.077			0.85 (0.75–0.96)	**0.011**

a OR, odds ratio.

b CI, confidence interval.

c OR estimated with logistic regression adjusted for sex and age.

### Dual luciferase reporter gene assays

**Figure 1:**
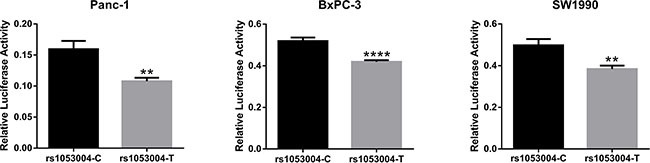
Dual luciferase reporter gene assays: the effects of rs1053004 on gene expression The figure showed that compared to the construct with rs1053004-T, the construct with rs1053004-C had significantly higher luciferase activity in three PC cell lines. Unpaired Student's *t*-test was used to evaluate the differences and *p* values less than 0.05 was considered significant with ** indicating *p* < 0.01 and **** indicating *p* < 0.001.

We constructed two luciferase reporter plasmids containing rs1053004 T and C allele, respectively. In all the three PC cell lines, Panc-1, BxPC-3 and SW1990, luciferase activity was significantly higher in the C allelic plasmid compared with the T plasmid (*P* < 0.05, Figure 1). The results suggested that rs1053004 T > C in 3′UTR could up-regulate gene expression post-transcriptionally.

## DISCUSSION

STAT3 plays a pivotal role in a multitude of physiological and pathological processes [31–36]. Its abnormal activity and expression have been found in PC [24, 29, 30, 37]. Nevertheless, whether genetic variations in this gene affect PC risk has been barely investigated. In our current study, using two stage case-control studies integrating luciferase reporter gene assays, we found for the first time that rs1053004 T > C in 3′UTR of *STAT3* might decrease risk of PC through up-regulating *STAT3* expression.

STAT3, a member of a transcription factor family, was first identified in 1994 as an IL-6-activated acute-phase response factor (APRF) [38]. Other from its transient activation in normal cells, *STAT3* was often found aberrantly expressed and constitutively activated in a variety of malignancies [39], thereby was regarded as an oncogene in multiple cancers [20–23]. Nonetheless, the tumor suppressing role of STAT3 was also reported [25–28, 40, 41]. For example, STAT3 was found as a negative regulator of thyroid cancer since it could activate transcription of the tumor suppressor insulin-like growth factor binding protein 7 (IGFBP7), and negatively regulate aerobic glycolysis [26]. In the context of lung cancer, STAT3 prevented disease initiation by maintaining pulmonary homeostasis under oncogenic stress [28]. Besides, loss of IL-6/Stat3 signaling in prostate cancer might bypass senescence and accelerates cancer progression via disrupting the ARF-Mdm2-p53 tumor suppressor axis [27]. In addition to these malignancies, other cancers were reported to benefit from STAT3 in phenotype and prognosis as well, such as breast cancer [40, 42], head and neck neoplasms [25], and brain tumor [43, 44]. In respect of PC, *STAT3* was suggested to be frequently over-expressed *in vivo* and vitro [24, 29, 37] and play a pivotal role in the carcinogenesis of PC, while its anti-tumor effect in PC is scarcely reported so far.

rs1053004 lies in the 3′UTR of *STAT3*, the mutant C allele is predicted to cause binding site loss for some miRNAs (such as hsa-miR-31-5p and hsa-miR-99b-3p, predicted by MicroSNiper web tool [45]). Consistently, dual luciferase reporter gene assays demonstrated in three different PC cell lines that C allele was significantly associated with higher luciferase activity. We therefore inferred that, in Chinese population, the C allele of rs1053004 astricted the binding of miRNA to *STAT3*, thus increasing the expression of this gene with potential benefit, and consequently conferred individuals with CC genotype lower risk to PC compared with TT genotypes, notwithstanding the exactly anti-PC mechanism of STAT3 still needs further investigation.

To the best of our knowledge, this is the first study to investigate the association between genetic variants in *STAT3* and risk of PC. Our work has three major strengths. First, two stage case-control studies with large sample size offer us enough statistical power to identify the association. Second, functional validation experiments not only indicated that the association might be reliable, but also provided the plausible underlying mechanisms of how this variant influenced risk of PC. Last but not least, our work suggests that *STAT3* might exert positive effects in preventing PC, particularly in Chinese population, despite the stereotype that *STAT3* was an oncogene. However, functional experiments such as RNA interference were still needed for deeper mechanism exploration. Besides, environmental factors such as smoking and obesity were not included in our current study. Further researches concerning gene-environment interaction will be required.

In conclusion, we identified a regulatory SNP rs1053004 mapping to 3′UTR of *STAT3* associated with a decreased risk of PC in Chinese population. Our findings could lead to new insights to the etiology of PC and provide a potential biomarker. Future studies conducted in different population and with deeper biological experiments are warranted to validate our results and more attention should be paid to the variants in *STAT3* when investigating genetic susceptibility to PC.

## MATERIALS AND METHODS

### Study subjects

We carried out a two-stage case-control study to investigate the association between variants of *STAT3* and risk of PC. Stage one included 774 cases and 777 cancer-free controls, part of which were also involved in our previous studies [46–49]. Patients were consecutively recruited from January 2009 to September 2014 at Tongji Hospital of Huazhong University of Science and Technology, Wuhan, China. While controls were volunteers randomly selected from healthy screenings over the same period. In stage two, 940 cases were enrolled at the Peking Union Medical College Hospital, Beijing from January 2008 to December 2012, and 1398 controls were cancer-free individuals from a community cancer screening program for early detection from the same region over the same period. All cases were individuals with newly diagnosed, pathologically confirmed and previously untreated primary PC. The diagnosis of PC was confirmed by histopathological or cytological analysis according to the World Health Organization classification. Controls were frequency matched with cases by sex and age (interval of 5 years). Demographic characteristics (sex, age) were obtained from medical records. At recruitment, a 2-mL peripheral venous blood sample was collected from each subject with informed consent. This study was approved by the ethics committee of Tongji Medical College of Huazhong University of Science and Technology and Peking Union Medical College Hospital.

### SNP selection genotyping and statistical analysis

It was primarily acknowledged that the 3′UTR often contains regulatory elements that post-transcriptionally regulate gene expression. For example, miRNAs usually interact with 3′UTR of target mRNAs leading to mRNA degradation and/or translational repression [50]. Accordingly, our hypothesis was that the variants in 3′UTR of STAT3 might affect its expression and further concern PC risk. We utilized the University of California Santa Cruz (UCSC) Table Browser (http://genome.ucsc.edu/cgi-bin/hgTables) to retrieve variants in 3′UTR of STAT3, finding four SNPs (rs1053004, rs1053005, rs1053023 and rs3744483) with minor allele frequency greater than 0.05. Among which, rs1053005, rs1053023 and rs3744483 are in perfect linkage disequilibrium (LD) (r^2^ = 1, using 1000 genome phase 3 data). While the LD relations between rs1053004 and others are slightly weaker (r^2^ = 0.8). Then we utilized SNPinfo Web Server (http://snpinfo.niehs.nih.gov/snpinfo/snpfunc.htm) to predict the function of these polymorphisms. All of the four SNPs lie in putative microRNA targets, and rs1053005 is predicted to regulate more microRNAs′ binding than the other two SNPs in perfect LD (rs1053023 and rs3744483). Taken together, the results of LD analysis and functional prediction suggested both rs1053004 and rs1053005 as most promising SNPs, and to be subsequently genotyped.

### Genotyping and statistical analysis

Genomic DNA was extracted from peripheral blood using RelaxGene Blood DNA System (Tiangen, DP319) according to the manufacturer's instructions. Quantitative and qualitative DNA analysis was performed with Nanodrop.

The two candidate SNPs were genotyped using a TaqMan assay on the ABI PRISM 7900 HT platform (Applied Biosystems, Inc.) in two stages. Genotyping analysis was conducted with Sequence Detection System (SDS) Software v2.4.1. For quality control, 5% duplicate samples were independently reanalyzed in a blinded fashion. The call rate of candidate SNPs were over 95%.

Deviation from Hardy-Weinberg equilibrium (HWE) in controls was examined by a goodness-of-fit χ^2^ test. Demographic characters and genotype frequencies differences were assessed by Pearson'sχ^2^ test. Odds ratios (ORs) and 95% confidence intervals (CIs) were calculated applying unconditional logistic regression analysis adjusting for age and sex. For all analyses, statistical significance was set at *P* < 0.05, and all tests were two sided. All statistical analyses were conducted by SPSS Statistics 20.0.

### Dual luciferase reporter gene assays

We chose psiCHECK™-2 Vector (Promega) to construct luciferase reporter gene plasmid since it is an ideal biosensor for miRNA-target interaction. This vector contains two kinds of luciferase gene, *Renilla* and *Firefly*. The former's activity reflected *STAT3* expression, as the 3′ UTR of which was appended to the *Renilla* luciferase gene. Inclusion of the firefly luciferase reporter served as the internal control. The whole 3′UTRs of STAT3 containing different alleles of SNP were cloned into the vectors (Genewiz). The reporter constructs were verified for sequence. Human pancreatic cancer cell lines Panc-1, BxPC-3 and SW1990 were used for luciferase reporter gene assays. The constructed reporter plasmid was transfected to the cells cultured in Dulbecco's Modified Eagle Medium (DMEM) with high glucose (Gibco) supplemented with 10% fetal bovine serum (Gibco) and 1% Penicillin-Streptomycin (Gibco) under 37°C in humidified atmosphere containing 5% CO2 using Attractene Transfection Reagent (QIAGEN). After 24 hours' incubation, the cells were assayed for two luciferase activity with the Dual-Luciferase Reporter Assay System (Promega). Relative activities were evaluated by calculating the ratio of Renilla to Firefly. For each plasmid, three independent transfection experiments were performed with each in triplicate. The differences in reporter gene expression were examined by Student's *t*-test.

## SUPPLEMENTARY MATERIALS TABLES


