# Supplementary material for: A single nucleotide polymorphism in the 3′-UTR of STAT3 regulates its expression and reduces risk of pancreatic cancer in a Chinese population
Source: Oncotarget. 2016 Aug 25;7(38):62305–11. doi: 10.18632/oncotarget.11607 (PMC5308728; doi:10.18632/oncotarget.11607)
Supplement: Supplementary file 1 [file oncotarget-07-62305-s001.pdf]

A single nucleotide polymorphism in the 3′-UTR of *STAT3* regulates its expression and reduces risk of pancreatic cancer in a Chinese population

Supplementary Materials

Supplementary Table S1: Association between rs1053005 and risk of PC

Genotype	Stage one			*P*
	Case *N* (%)	Control *N* (%)	ORa (95% CI[b])[c]	
rs1053005				
AA	378 (48.8)	349 (44.9)	1.000	
AG	313 (40.5)	332 (42.7)	0.87 (0.70–1.08)	0.198
GG	83 (10.7)	96 (12.4)	0.80 (0.57–1.11)	0.176
Additive			0.89 (0.76–1.03)	0.105
Rscessive			0.85 (0.62–1.16)	0.314
Dominant			0.85 (0.70–1.04)	0.120

[a]OR, odds ratio.
[b]CI, confidence interval.
[c]OR estimated with logistic regression adjusted for sex and age.

Supplementary Table S2: Association between rs1053004 and risk of PC stratified by gender

gender	Stage one		Stage two		combined	
	OR (95% CI)[a]	*P*	OR (95% CI)[a]	*P*	OR (95% CI)[a]	*P*
Male	0.88 (0.73–1.07)	0.195	0.85 (0.74–0.98)	**0.023**	0.86 (0.77–0.96)	**0.009**
Female	0.80 (0.65–0.99)	**0.045**	0.89 (0.68–1.18)	0.419	0.85 (0.77–1.01)	0.057

[a]OR estimated with logistic regression adjusted for sex and age.
